# Effects of exposure to facial expression variation in face learning and recognition

**DOI:** 10.1007/s00426-014-0627-8

**Published:** 2014-11-15

**Authors:** Chang Hong Liu, Wenfeng Chen, James Ward

**Affiliations:** 1 Department of Psychology, Bournemouth University, Talbot Campus, Fern Barrow, Poole, BH12 5BB UK; 2 Institute of Psychology, Chinese Academy of Sciences, Beijing, China; 3 Department of Computer Science, University of Hull, Kingston upon Hull, UK

## Abstract

Facial expression is a major source of image variation in face images. Linking numerous expressions to the same face can be a huge challenge for face learning and recognition. It remains largely unknown what level of exposure to this image variation is critical for expression-invariant face recognition. We examined this issue in a recognition memory task, where the number of facial expressions of each face being exposed during a training session was manipulated. Faces were either trained with multiple expressions or a single expression, and they were later tested in either the same or different expressions. We found that recognition performance after learning three emotional expressions had no improvement over learning a single emotional expression (Experiments 1 and 2). However, learning three emotional expressions improved recognition compared to learning a single neutral expression (Experiment 3). These findings reveal both the limitation and the benefit of multiple exposures to variations of emotional expression in achieving expression-invariant face recognition. The transfer of expression training to a new type of expression is likely to depend on a relatively extensive level of training and a certain degree of variation across the types of expressions.

## Effects of exposure to facial expression variation in face learning

Along with pose and illumination, facial expression is a major source of image variation in face stimuli. It is well known that image variation can substantially impair recognition of unfamiliar faces (Hancock, Bruce & Burton, 2000). Prior research has focused on the effects of pose and illumination variation on face recognition (e.g., Johnston, Hill & Carman, 1992; Tarr, Georghiades & Jackson, 2008; Liu, Bhuiyan, Ward & Sui, 2009; Liu, Collin, Burton & Chaudhuri, 1999; Wallraven, Schwaninger, Schuhmacher & Bülthoff, 2002). In contrast, little research has investigated how observers handle expression variation. Given the deficit of recognising an unfamiliar face in a previously unseen expression (Bruce, 1982), the primary motivation of this study is to understand the minimal requirement for improvement or more robust, expression-invariant face recognition.

An obvious route to image-invariant face recognition is familiarisation through growing level of exposure. It is known that learning several poses of a face can facilitate pose-invariant recognition (Hill, Schyns & Akamatsu, 1997; Logie, Baddeley & Woodhead, 1987; Longmore, Liu & Young, 2008, 2014; Wallraven et al., 2002). This literature shows that improvement can be observed after a relatively brief training session that involves exposure to a small number of pose variations. Training of this kind can strengthen pose-invariant representations in the visual cortex (Eger, Schweinberger, Dolan & Henson, 2005; Pourtois, Schwartz, Seghier, Lazeyras & Vuilleumier, 2005). Following the methods used in these training studies, we investigated whether a similar manipulation of exposure to several expressions could improve expression-invariant recognition.

Image variation due to different sources may require different kinds of visual processing. The same processing strategies for variation due to pose or illumination may not be suitable for image variation due to facial expression. Research has shown that pose or viewpoint generalisation in both face and object recognition can be achieved via linear combination such as interpolation or extrapolation from a small number of stored views (Bülthoff & Edelman, 1992; Wallraven et al., 2002). For example, once a frontal pose and side pose of a face are learned, a range of other poses in between can be predicted through pose interpolation. Although no research to date has extended the theory of viewpoint generalisation to recognition of a face in a novel expression, it is possible that interpolation and extrapolation are also used in expression generalisation. However, these methods may not be useful for predicting variations of facial expression in the present study because the difference between expressions is often categorical or discrete, while viewpoint variation is often continuous. This means that a face with a happy expression may not be predicted from the same face studied in surprise and angry expressions, because the studied expressions do not form a continuum with the happy expression. Interpolation or extrapolation is unlikely to be suitable for image variation created by different categories of expression. Unless there are alternative processing methods, predicting a face with a categorically different type of expression from the stored expressions of the face can be difficult.

In this context, it is useful to distinguish two kinds of transfer in face learning. Generalisation from a learned expression may occur either within or across categories. Generalisation from a smiling to an angry face involves a between-category transfer because these expressions belong to different emotional categories. On the other hand, generalisation from a smiling to a laughing face can be considered as a within-category transfer because these are variants of the happy expression. Within-category variance can be roughly described by intensity that varies from a minimum to a maximum of that expression. We should note, however, within each basic category, there can be subordinate categories, which can contain further categorical boundaries and hence are not strictly quantitative. Nevertheless relative to the categorical boundary at an upper level, the difference within a class of expression may be less distinct. This may allow the room to characterise the within-category variation by intensity as a rough approximation. If such approximation is possible, then interpolation or extrapolation may be useful for handling within-category variation when two or more variants of the expression are stored in memory. However, because neither of these methods would be suitable for between-category transfer, one possibility is that expression-invariant recognition would require storing at least one instance for each category of expression. There is evidence why to some extent this could be true. Hay, Young and Ellis, (1991) have shown that even for well-learned familiar faces where recognition is typically expression invariant, unusual expressions can still slow down or hamper recognition performance. This suggests that expression-invariant recognition may require exposure to several categories of expression.

However, some degree of between-expression transfer may be expected because the visual system may use general knowledge of expressions and certain image-invariant features such as skin tones and textures. Burton, Jenkins, Hancock and White, (2005) have shown that averaging multiple images of a face can form a robust representation against a range of image variations including expression variation. This may suggest that although it is important to form a robust representation from multiple images, exposure to all types of expression is not necessary. However, because this line of research mainly employed expressions that are commonly found in the public media and Internet, the range of emotions and the differences between them in stored and test images can be limited. Like the vast majority of photographs, these images commonly show various smiling faces. Therefore, although exposure to a commonly seen expression such as smiling is useful for recognising the person with similar expressions in different images, it remains unclear whether this experience is equally useful for recognising the person with a quite different expression such as disgust or fear.

The existing theories or methods for dealing with image variation in face images have not been explicitly or systematically tested for their ability to account for expression-invariant face recognition. A main purpose of this research was to examine the implications of these current theories of face recognition in predicting between-category transfer of expression training. If linear combination by interpolation or extrapolation is the only available mechanism for predicting a new image, it should be very difficult to predict and recognise a face in a new expression based on some previously studied images of the face that showed categorically different expressions. There should be no difference in the results of recognition performance whether a single or several such expressions of the face are learned. On the other hand, if expression-invariant recognition depends on the same underlying principle for other kinds of image-invariant recognition, then the key to achieving expression-invariant recognition should be to maximise the exposure to image variation, locating the corresponding features in different images, and forming an average representation across different instances of the learned face. This would predict a better generalisation to a new expression after several other expressions of the face are learned. There is some evidence that exposure to just two different images of a face can improve sequential matching performance (Menon, Kemp & White, 2013). In the present study, we attempted to determine whether a similar benefit of exposure to multiple images could be observed in a long-term memory task.

A major manipulation in this study, therefore, was the level of exposure to expression variation. We assessed whether studying three expressions of a face is enough to facilitate recognition of the studied face with a new expression. We measured the potential benefit of this by comparing the recognition performance with a baseline condition where a single facial expression of the face was studied. We conducted three experiments; each compared the same multiple-expression training condition to a different baseline. The baseline in Experiment 1 was an emotional expression with three levels of intensity. Experiment 2 also used an emotional expression but without variation of intensity. Finally, Experiment 3 used a neutral expression.

## General method

Because face learning involves storing facial information in long-term memory, we employed the standard old/new recognition task. Participants were required to remember faces in a training session and later identify them in a test session. Participants were randomly assigned to one of the two training conditions, where each face was either learned through three expressions or one expression. Here, we define the identity of this learned face as a “target”. In the test session, the target faces were mixed with new identities of faces that were not shown in the learning session. We defined the new faces as “distractors”. The exact facial expressions of the target faces could vary between learning and test sessions. Because faces are not equally easy to remember, we presented the same faces to both groups. However, instead of using the same targets/distractors assignments in the same order for all participants, we randomly assigned the faces to targets/distractors and randomised the order of these for every two participants, one from each condition. This guaranteed that both participants saw the same set of target and distractor faces in the same order, and the only difference between them was that one participant learned three expressions, whereas the other participant learned just one expression of the target faces during the training session.

### Materials

**Fig. 1:**
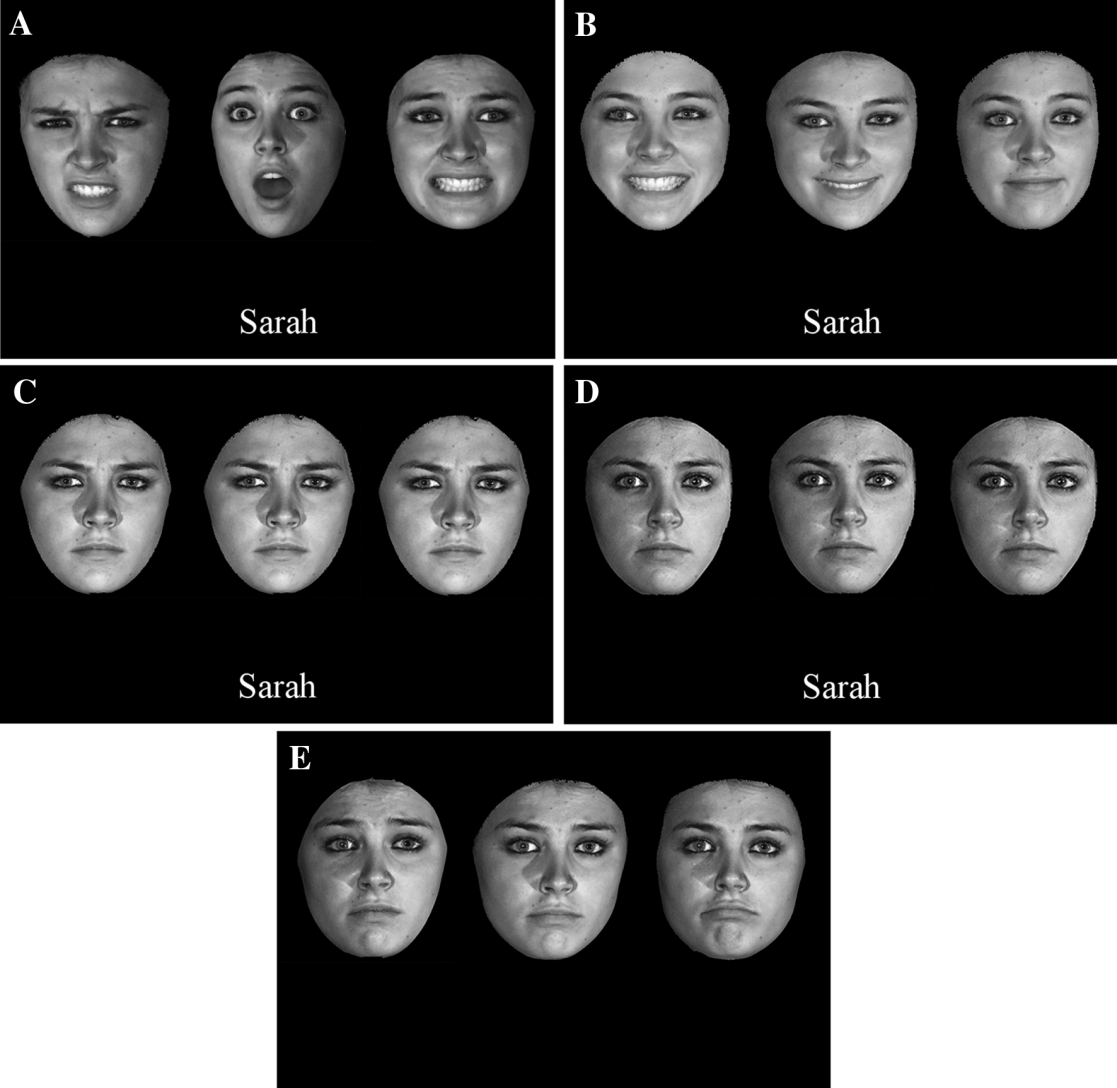
Examples of a learn trial in the face-name presentation session. **a** Multiple-expression training in all three experiments: a face is shown in three randomly chosen categorically different expressions: Disgust, Surprise and Fear. **b** Single-expression training in Experiment 1: The same face is shown in a randomly chosen expression of three varying strengths. The emotional strengths of the three images from the *left* to *right* were at levels of 3, 2, and 1, where 1 represents the weakest strength of the happy expression. **c** Single-expression training in Experiment 2: The level of emotional strengths of the three images had the identical strength of 4 of the sad expression. **d** Single-expression training in Experiment 3: All three images had the identical neutral expression. **e** An example of a test trial in Experiment 1. The emotional strengths of the three images from the *left* to *right* were levels 3, 1, and 2 of the sad expression. In Experiments 2 and 3, the three test images were identical to one another

We used a 3D face database from Binghamton University (Yin, Wei, Sun, Wang and Rosato, 2006). It contained 100 faces without facial hair or spectacles. All faces were captured in seven different expressions: neutral, happy, sad, angry, fear, disgust and surprise. Each emotional expression was also captured in four levels of intensities. We used only the strongest intensity for all our conditions except for the baseline condition in Experiment 1 where a variety of intensity was used. A pool of 30 female Caucasian faces was chosen from the database. All faces were shown in a full frontal view. Images were scaled to 220 × 220 pixels, which measured 13.6 × 10.2º of visual angle at the viewing distance of 60 cm. All images were shown in black and white with 256 levels of grey, displayed against a uniform black background. An example face is shown in Fig. 1.

A total of 12 ordinary first English names were used in the face-name learning session. The names ranged from four to six characters long, e.g., Jane, Rose, and Sarah.

The stimuli were displayed on a 21″ monitor (SONY Trinitron, GDM-F520), with a screen resolution of 1024 × 768. The vertical frequency of the monitor was 120 Hz. The study was run on a Pentium 4 computer. The software for experimental control was written in MATLAB 6.5 for PC, with Psychophysics Toolbox extensions (Brainard, 1997; Pelli, 1997).

### Assignment of face stimuli

A total of 24 individuals’ faces from the pool were randomly chosen for every pair of participants, one from the multiple-expression condition and the other from the single-expression condition. Half of these 24 faces were randomly assigned as targets and the remaining 12 as distractors.

#### Multiple-expression condition

In the training sessions, each of 12 target faces was assigned three emotional categories, which were randomly selected from the six emotional categories (an example is given in Fig. 1a). In the following, we use the terms “emotional categories”, “emotional expressions”, and “expressions” interchangeably to denote different emotional categories of expressions that could or could not be of the same intensity. If we refer to different intensities of the same emotional expression, we will explicitly say so. The random assignment followed the constraint that each emotional expression was selected an equal number of times for the target faces. Because a total of 36 images were used for training (12 faces × 3 expressions), each emotional expression was used exactly six times (36 images/6 emotional categories).

In the test session, 6 of the 12 target faces were randomly assigned a new expression of 3 varying intensities, randomly chosen from the 4 available levels of intensity. The remaining six target faces were shown in images identical to the training sessions. The method for creating the distractor faces was identical to that for creating the target faces in the test session.

#### Single-expression condition

In training sessions of Experiments 1 and 2, each of the 12 target faces was randomly assigned a single expression from the six emotional expressions. Each emotion was assigned exactly twice for the 12 target faces. In Experiment 1, the assigned expression was shown in three images of different emotional intensities, randomly from the four levels (an example is given in Fig. 1b). In Experiment 2, the assignment of the emotional expressions to the target faces was the same except that the three images used for each face had the identical highest level of emotional intensity (i.e., the three images were identical, as in the example shown in Fig. 1c). In Experiment 3, all target faces in the training sessions were assigned a neutral expression. The neutral face was also shown in three identical images (Fig. 1d).

In the test session, we used the same method as that for the multiple-expression condition. That is, we randomly assigned 6 of the 12 target faces to a new emotional expression. In Experiment 1, this had three varying intensities (Fig. 1e), whereas in Experiments 2 and 3, the intensity for a new expression was constant, randomly chosen from the four levels for each face. The remaining 6 target faces were shown in images identical to the training sessions. Again, the method for creating the distractor faces was identical to that for creating the target faces in the test session.

### Procedure

Past research shows that learning a face only once either through a single image or multiple images is often insufficient to produce a transfer of training (Liu et al., 2009; Longmore et al., 2008). A transfer to a new image may be achieved, however, when a more robust representation of the trained images is formed. Following the method in these studies, we employed a face-name matching procedure to engage participants in the learning process. This required participants to pair names with faces, which allowed each trained face to be viewed several times before the recognition test session. The required level of training was determined by a pilot study, which showed that each face should be shown at least four times during the training session to reach a recognition performance level between floor and ceiling. All experiments consisted of a face-name presentation session, a face-name training session, and a test session.

#### Face-name presentation session

In this initial session, the participant was presented with 12 target faces, one at a time, for 5 s. Each face was presented in a row of three images in the centre of the screen, where the margin on the left and right of the stimuli was 8.4º, whereas the margin from the top and bottom was 16.9º of visual angle. In the multiple-expression condition, the three images displayed three different emotions. In the single-expression condition, the three images either showed an emotional expression with three levels of intensity (Experiment 1), or duplicates of an emotional expression with the identical intensity (Experiment 2), or duplicates of a neutral expression (Experiment 3). In both conditions, a name was presented simultaneously below each face. The assignment of the names to faces was random. Participants were instructed to memorise the pair of face and name.

#### Face-name training session

Immediately after the face-name presentation session, each learned face was shown again as in the previous session. This time the face was paired with a row of four names at the bottom of the screen. One of the names had been paired previously with the face. The others were randomly chosen from the 12 names. The order of the names on the screen was random. The task was to indicate which name was associated with the face by pressing one of the four corresponding keys. Feedback is given following the participant’s response. The correct answer was shown when a wrong name was chosen. The block of face-name matching trials was repeated three times for all participants regardless of their performance on the face-name matching task.

#### Test session

The recognition test followed immediately after the face-name training session. Here the trained faces were presented with 12 distractor faces, again one at a time. The order of presentation of target faces during the testing session was the same as in the final set of the training session, but distractor faces were randomly inserted into the sequence between targets. We did not randomise the order of the target faces again after the final training session, because doing so could accidentally present the target face at or near the final trial of training session at the beginning of the test session. This could introduce an undesirable recency effect. The names were not presented in the test session. The trained faces were either shown in identical images as the two training sessions or in a novel expression. Each emotional expression was shown twice, once as a target and once as a distractor. Participants were asked to decide whether each test face had been shown at the learning session. They pressed the key labelled “Yes” if the face was seen during the learning session or the key labelled “No” otherwise.

### Design

The transfer from training to a new expression was assessed in a 2 × 2 mixed design. The between-participants variable was level of exposure (multiple vs. single), and the within-participants variable was test expression (same vs. different). The dependent variables were sensitivity (*d*′) and criterion (*c*) that combined hits and false alarms.

## Experiment 1

The purpose of this experiment was to investigate whether learning three different emotional expressions of a face can transfer better to a new emotional expression than learning a single expression of varying intensity. The experiment tested the hypothesis that a between-expression transfer is more likely to benefit from exposures to cross-expression variation rather than within-expression variation.

### Participants

A group of 80 students (65 females) from the University of Hull were randomly assigned to the two conditions. Each condition had 40 participants whose ages ranged from 18 to 35 (Mdn = 19). All had normal or corrected-to-normal vision. Written informed consent was obtained from all participants.

### Results and discussion

#### Training session results

The face-name matching data of the two training conditions over the three training blocks were analysed using a 2 × 3 repeated-measures ANOVA. The accuracy measure was the percentage of correctly matched face-name pairs out of the total 12 pairs. Given the four-alternative forced choice task, the chance level performance was 25 %. The mean accuracy was 47.9 % (SD = 14.3) for participants trained in three different emotional expressions, and 52.9 % (SD = 14.3) for participants trained in a single expression of varying intensity. This difference was not statistically significant, *F* (1, 78) = 2.45, partial *η* ^2^ = 0.03, *p* = 0.12. The mean matching performances from block 1 to 3 were 48.0 % (SD = 17.1), 50.2 % (SD = 16.8), and 53.0 % (SD = 19.3), respectively. The main effect showed significant improvement over the three blocks, *F* (2, 156) = 3.04, partial *η* ^2^ = 0.04, *p* = 0.05. There was no interaction between the type of exposure and training blocks, *F* (2, 156) = 0.30, partial *η* ^2^ ~ = 0.00, *p* = 0.74.

#### Test session results

**Fig. 2:**
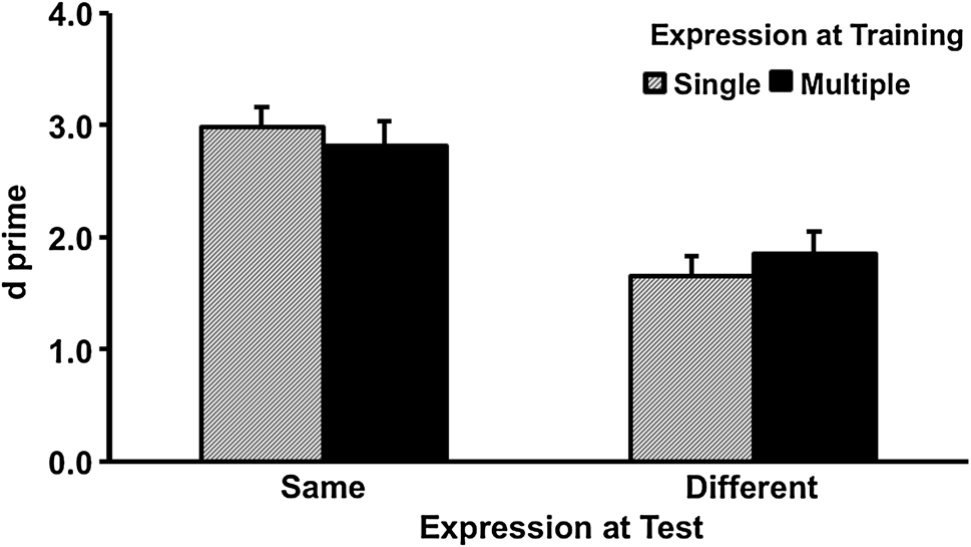
Accuracy as a function of expression training and expression change in Experiment 1. *Error bars* represent one standard error above the means

Figure 2 shows the *d*′ results of the recognition test. ANOVA found no effect of exposure, *F* (1, 78) = 0.01, partial *η* ^2^ ~ = 0.00, *p* = 0.94. Recognition performance in the same expression condition (*M* = 2.90, SD = 1.24) was better than the different expression condition (*M* = 1.75, SD = 1.21), *F* (1, 78) = 45.76, partial *η* ^2^ = .37, *p* < 0.001. The interaction between exposure and expression change was not significant, *F* (1, 78) = 1.19, partial *η* ^2^ = 0.02, *p* = 0.28.

The criterion data are shown in Table 1. The main effect of expression training was not significant, *F* (1, 78) = 0.14, partial *η* ^2^ ~ = 0.00, *p* = 0.71. The criterion results for the same expression (*M* = −0.37, SD = 0.56) were more liberal than for the different expression condition (*M* = 0.14, SD = 0.61), *F* (1, 78) = 41.98, partial *η* ^2^ = 0.35, *p* < 0.001. The interaction between the two factors was also significant, *F* (1, 78) = 5.37, partial *η* ^2^ = 0.06, *p* = 0.02. The interaction was due to a greater difference between the criterion results of the single-expression compared to multiple-expression training. The difference between criteria in each training group was calculated by subtracting criterion for different expression from that for same expression. The mean differences for the single-expression and multiple-expression training groups were 0.69 (SD = 0.77) and 0.33 (SD = 0.38), respectively, *t* (78) = 2.32, *p* = 0.02.

**Table 1:** Criterion results (c) as a function of training and test conditions in Experiment 1

Training condition	Expression at test			
	Same		Different	
	*M*	SD	*M*	SD
Multiple expression	−0.30	0.54	0.03	0.59
Single expression	−0.44	0.57	0.25	0.62

This experiment showed a typical expression-dependent effect in face recognition, where a change of expression from learning to test affected both sensitivity and response criterion. The face-name matching results showed expected improvement over the course of training in matching repeatedly shown identical face images with the correct names. The key finding, however, was that learning three categorically different expressions of a face was not more useful than learning three different images of the same expression. The two conditions created comparable recognition performance for faces tested in a new expression. However, it is possible that learning image variation within the same category of an emotional expression also had some facilitating effect on transfer to a new facial expression. Comparing a potential benefit of multiple-expression training with a baseline that may have a benefit itself could make the test less sensitive. To increase the sensitivity of the test, we decided to remove the image variation in the single-expression training condition in the next experiment.

## Experiment 2

In this experiment, our purpose was again to assess whether learning three different emotional expressions of a face allows better between-expression transfer compared to learning one expression. We employed the same design as Experiment 1. However, instead of using three different levels of emotional intensity in the single-expression condition, we used three identical images of an emotional expression for this condition. Again, we tested the hypothesis that exposure to three different expressions of a face should result in a better between-expression transfer than exposure to a single expression of the face.

### Participants

A different group of 81 students (ages ranged from 15 to 35, Mdn = 20; 56 female) from the University of Hull were randomly assigned to the two conditions. The single-expression and multiple-expression conditions had 40 and 41 participants, respectively. All had normal or corrected-to-normal vision. Written consent was obtained from all participants.

### Results and discussion

#### Training session results

Participants in the multiple-expression training condition scored lower face-name matching performance (*M* = 49.7 %, SD = 12.7) relative to those in the single-expression training condition (*M* = 61.3 %, SD = 15.0), *F* (1, 79) = 14.00, partial *η* ^2^ = 0.15, *p* < 0.001. The matching performance improved over the three blocks, where the means for the two groups from block 1 to block 3 were 52.0 % (SD = 16.9), 54.4 % (SD = 18.9), and 59.9 % (SD = 19.6), respectively, *F* (2, 158) = 7.35, partial *η* ^2^ = 0.09, *p* < 0.01. The interaction between the two factors was not significant, *F* (2, 158) = 0.10, partial *η* ^2^ ~ = 0.00, *p* = 0.91.

#### Test session results

**Fig. 3:**
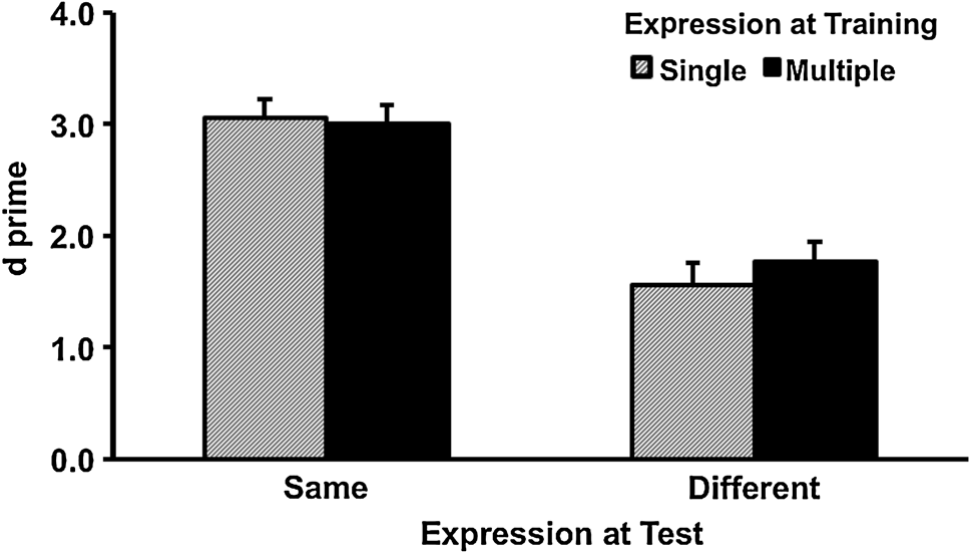
Accuracy as a function of expression training and expression change in Experiment 2. *Error bars* represent one standard error above the means

The *d*′ results of the recognition test are shown in Fig. 3. Faces trained in multiple expression (*M* = 2.39, SD = 1.11) and single expression (expression (*M* = 2.31, SD = 1.12) created comparable performance, *F* (1, 79) = 0.12, partial *η* ^2^ ~ = 0.00, *p* = 0.73. Faces tested in the same expression (*M* = 3.03, SD = 1.03) as the training session created better performance than in a different expression (*M* = 1.66, SD = 1.18), *F* (1, 79) = 110.43, partial *η* ^2^ = 0.58, *p* < 0.001. The interaction between these factors was not significant, *F* (1, 79) = 0.98, partial *η* ^2^ = 0.01, *p* = 0.33.

Table 2 shows the criterion results. The criterion for faces trained and tested in the same expression (*M* = −0.51, SD = 0.46) was more liberal than the criterion for faces trained and tested in different expression (*M* = 0.24, SD = 0.63), *F* (1, 79) = 101.49, partial *η* ^2^ = 0.56, *p* < 0.001. Criterion for the multiple-expression training (*M* = −0.14, SD = 0.55) was comparable to criterion for the single-expression training (*M* = −0.13, SD = 0.54), *F* (1, 79) = 0.01, partial *η* ^2^ = 0.01, *p* = 0.92. The interaction between these was not significant, *F* (1, 79) = 0.68, partial *η* ^2^ = 0.01, *p* = 0.41.

**Table 2:** Criterion results (c) as a function of training and test conditions in Experiment 2

Training condition	Expression at test			
	Same		Different	
	*M*	SD	*M*	SD
Multiple expression	−0.48	0.46	0.20	0.65
Single expression	−0.53	0.47	0.27	0.62

This experiment again showed typical expression-dependent effects of expression change with reduced sensitivity and conservative response criterion. The reduced face-name matching performance in the multiple-expression training condition relative to the single-expression condition indicated greater demand in learning to associate three different face images to the same name. However, the key finding was that recognition performance in both the multiple and single-expression training conditions dropped to a similar level when the expression of the learned faces was changed in the test session. Consistent with Experiment 1, this again suggests that exposure to three expressions of a face does little to improve expression-invariant face recognition.

In both Experiments 1 and 2, the potential benefit of exposure to multiple emotional expressions was measured against the result of exposure to a single emotional expression. However, there is a possibility that the benefit of the multiple-expression training partially relies on the expressions being emotional. If so, because the baseline condition also employed an emotional expression, the chance of detecting the small benefit could have been weakened. To test this possibility, we used neutral faces in the baseline condition of our next experiment.

## Experiment 3

Both Experiments 1 and 2 were unable to demonstrate advantage of exposure to three expressions of a face relative to one expression in between-expression transfer. In this experiment, we tested the possibility that the potential advantage of three-expression training is more detectable when the result is compared to a baseline using a neutral expression.

### Participants

A different group of 83 students (56 females) from the University of Hull were randomly assigned to the two conditions. The multiple-expression training group had 42, whereas the single-expression training group had 41 participants. The ages of the participants ranged from 18 to 50 (Mdn = 20). All had normal or corrected-to-normal vision. Written consent was obtained from all participants.

### Results and discussion

#### Training session results

The participants in the multiple-expression training condition produced poorer face-name matching performance (*M* = 48.7 %, SD = 13.0) relative to those in the single-expression training condition (*M* = 57.3 %, SD = 15.0), *F* (1, 81) = 7.88, partial *η* ^2^ = 0.09, *p* < 0.01. There was also a significant main effect of training repetition, *F* (2, 162) = 5.83, partial *η* ^2^ = 0.07, *p* < 0.01, where face-name matching performance improved over the three training blocks. The mean matching accuracies in block 1 through 3 were 50.1 % (SD = 19.4), 51.9 % (SD = 16.7), and 57.1 % (SD = 18.9), respectively. The interaction between the two factors was not significant, *F* (2, 162) = 0.08, partial *η* ^2^ ~ = 0.00, *p* = 0.93.

#### Test session results

**Fig. 4:**
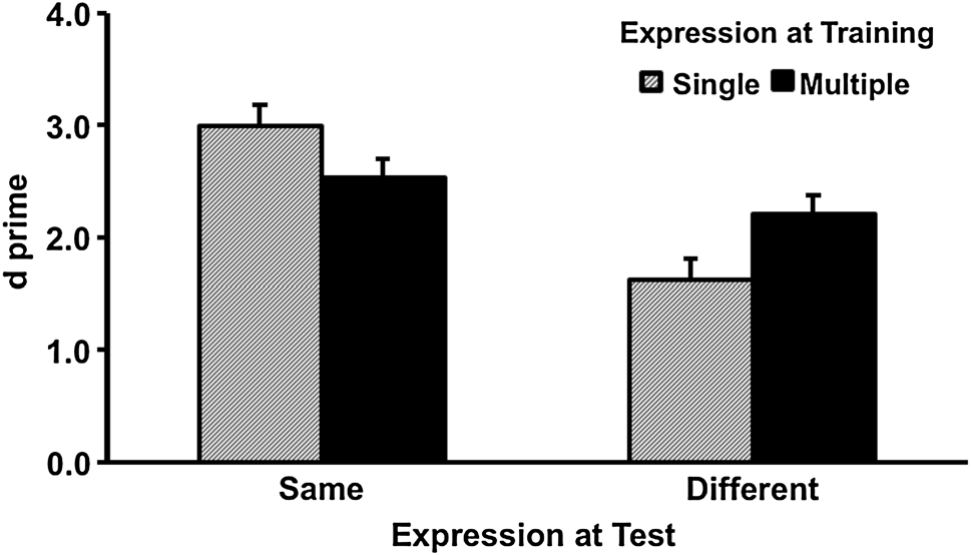
Accuracy as a function of expression training and expression change in Experiment 3. *Error bars* represent one standard error above the means

The *d*′ results of the recognition test are shown in Fig. 4. The main effect of exposure was not significant, *F* (1, 81) = 0.08, partial *η* ^2^ = 0.00, *p* = 0.78. The main effect of expression change was significant, where recognition was more accurate when faces were trained and tested in the same expression (*M* = 2.76, SD = 1.16) rather than different expression (*M* = 1.92, SD = 1.17), *F* (1, 81) = 32.62, partial *η* ^2^ = 0.29, *p* < 0.001. Importantly, there was a significant interaction between training condition and expression change, *F* (1, 81) = 12.60, partial *η* ^2^ = 0.01, *p* < 0.01, which was caused by the relatively shallower drop of performance in the multiple-expression training condition when the expression was different between the training and the test sessions. Simple main effect analyses confirmed that when the trained faces were tested in the same expression, the performance in the multiple-expression training condition (*M* = 2.53, SD = 1.12) was comparable if not worse than the single-expression training condition (*M* = 3.00, SD = 1.18), *F* (1, 81) = 3.48, partial *η* ^2^ = 0.04, *p* = 0.07. In contrast, when the trained faces were tested in a different expression, the performance in the multiple-expression training condition (*M* = 2.21, SD = 1.12) was significantly better than the single-expression training condition (*M* = 1.63, SD = 1.16), *F* (1, 81) = 5.43, partial *η* ^2^ = 0.06, *p* = 0.02.

The criterion results are shown in Table 3. The main effect of training condition was not significant, *F* (1, 81) = 0.13, partial *η* ^2^ ~ = 0.00, *p* = 0.72. The criterion for faces trained and tested in the same expression (*M* = −0.52, SD = 0.49) was more liberal than for faces trained and tested in different expression (*M* = 0.03, SD = 0.75), *F* (1, 81) = 40.42, partial *η* ^2^ = 0.33, *p* < 0.001. The interaction between these two factors was not significant, *F* (1, 81) = 0.99, partial *η* ^2^ = 0.01, *p* = 0.32.

**Table 3:** Criterion results (c) as a function of training and test conditions in Experiment 3

Training condition	Expression at test			
	Same		Different	
	*M*	SD	*M*	SD
Multiple expression	−0.45	0.54	0.01	0.72
Single expression	−0.58	0.42	0.05	0.79

Unlike Experiments 1 and 2, the faces learned in this experiment were discriminated better in a new expression following the multiple-expression training. The results suggest that repeated exposures to three categorically different expressions of a face could create a better transfer to a new expression relative to exposure to a neutral face.

### Cross-experiment comparison

Were the results in Experiment 3 fundamentally different from the other two experiments? To understand whether the pattern of the *d’* results differed among the three experiments, we performed a three-way repeated-measures ANOVA, using experiment as an additional between-participant factor. If the better between-expression transfer were unique to Experiment 3, we would expect a significant three-way interaction among experiment, exposure, and expression change, where only Experiment 3 should display better transfer to a new expression following multiple-expression training.

The ANOVA showed no main effects of experiment, *F* (2, 238) = 0.01, partial *η* ^2^ = 0.00, *p* = 0.99, or level of exposure, *F* (1, 238) = 0.16, partial *η* ^2^ ~ = 0.00, *p* = 0.68. Recognition was impaired when learned faces were tested in a different expression relative to the same expression, *F* (1, 238) = 167.38, partial *η* ^2^ = 0.41, *p* < 0.001. There was no three-way interaction, *F* (2, 238) = 2.07, partial *η* ^2^ = 0.02, *p* = 0.13, or two-way interaction between exposure and experiment, *F* (2, 238) = 0.02, partial *η* ^2^ ~ = 0.00, *p* = 0.98. However, the results were qualified by two-way interactions between exposure and expression change, *F* (1, 238) = 10.43, partial *η* ^2^ = 0.04, *p* < 0.01, and between experiment and expression change, *F* (1, 238) = 3.09, partial *η* ^2^ = 0.03, *p* < 0.05.

The interaction between exposure and test expression was due to a better transfer of training to a new expression following exposure to three expressions relative to a single expression. When trained faces were tested in the same expression, the results for three-expression (*M* = 2.78, SD = 1.19) and single-expression (*M* = 3.01, SD = 1.10) were comparable, *t* (242) = −1.59, *p* = 0.11. However, when studied faces were tested in a new expression, the result for three-expression (*M* = 1.94, SD = 1.41) was significantly better than single-expression (*M* = 1.61, SD = 1.35), *t* (242) = 2.20, *p* < 0.03.

To identify the source of interaction between experiment and expression change, we first conducted simple main effects analyses for same and different expression separately. The results showed that when faces were tested with the same expression as the trained, there was no difference among the three experiments, *F* (2, 241) = 1.15, partial *η* ^2^ ~ = 0.00, *p* = 0.32. There was also no difference among the experiments when the trained faces were tested in a different expression, *F* (2, 241) = 0.99, partial *η* ^2^ = 0.01, *p* = 0.37. As our analysis for each individual experiment already indicated, there was a strong effect of expression change in all experiments. However, effects of this varied across the three experiments. We calculated the effect of expression change in each experiment by subtracting the *d′* for different expression from the *d′* for same expression. The effects for Experiments 1 through 3 were 1.15 (SD = 1.52), 1.37 (SD = 1.17), and 0.84 (SD = 1.44), respectively. An ANOVA showed a significant difference among these, *F* (2, 241) = 3.01, partial *η* ^2^ = 0.01, *p* = 0.05. A Tukey pairwise comparison of means showed that the change of expression had significantly less impact in Experiment 3 compared to Experiment 2. Other pairwise comparisons did not find significant difference. The interaction between experiment and expression change was, therefore, due to relatively smaller effect of expression change in Experiment 3.

The cross-experiment analysis showed that multiple-expression training in all experiments created similar effects relative to single-expression training. This was evident in the two-way interaction between exposure and expression change. In other words, the analysis showed no evidence that only the baseline condition in Experiment 3 of this study resulted in the interaction. The analysis merely showed that the effect of expression change was smaller in Experiment 3 than the other two experiments. The lack of three-way interaction or two-way interaction between level of exposure and experiments suggests that both multiple-expression training and single-expression training conditions produced comparable recognition performance across the three experiments.

## General discussion

The primary aim of this study was to evaluate whether exposure to three categorically different expressions of a face is sufficient to facilitate subsequent recognition of the studied face with a new expression. We compared the results of this with different baseline conditions in three experiments. Recognition showed little evidence of improvements relative to the exposure to a single emotional expression, whether the single expression had different levels of intensity (Experiment 1) or fixed intensity (Experiment 2). However, an improvement was observed when results of multiple-expression training were compared to the exposure to a neutral expression (Experiment 3).

Although results from Experiments 1 and 2 showed no significant advantage of exposure to three expressions over the single-expression training baseline, our across-experiments analysis suggests that the effect of exposure to three expressions may not differ qualitatively across experiments. The discrepancy in these analyses is likely to be explained by increased power in the combined data set. Given the already fairly large sample size (*N* ≥ 80) in each experiment, it shows the transfer effect following three-expression training is small and hard to detect.

The transfer effect following multiple-expression training is not easily explained by any theory relying on a linear combination of images. Although interpolation and extrapolation are suitable for predicting the benefit of pose training, it is difficult to see how these putative mechanisms predict the observed transfer of expression training. If the learned expressions of a face were categorically different from the expression of the face at the test session, it would not be possible to predict the test face by combining the stored images. Prior studies on pose training have shown that although transfer to a new pose can be achieved by interpolation or extrapolation of the learned poses, these processing methods are not very useful if the new pose is orthogonal to the learned poses (Bülthoff & Edelman, 1992; Wallraven et al., 2002).

The facilitating effect of encoding multiple images of a face may be better explained by the benefit of averaging. A representation averaged over multiple images of a face is more resilient to various image variations including facial expression (Burton et al., 2005; Burton, Jenkins & Schweinberger, 2011). Averaging relies on knowing that several different face images are of the same person. By showing three face images with different expressions side by side and by identifying them as the same person, the training procedure should have facilitated the hypothetical averaging process. Menon et al., (2013) have shown that even limited exposure to just two different images of a face can improve sequential matching performance. White, Burton, Jenkins and Kemp, (2014) have further demonstrated that multiple images of a person or an average of multiple images of the face enjoy an advantage in a simultaneous matching task relative to a single image of the face. The present study has shown that a similar benefit of multiple exposures can also be demonstrated in a long-term memory task, although this requires far greater level of exposure with more image variation and repetitions.

It is worth noting, however, that averaging or exposure to a number of images alone cannot provide specific prediction about recognition performance. An important factor is the types of variation in the studied images. Transfer between types of image variation is more difficult than within a type of variation due to lower degree of image similarities. Prior research has shown that certain kinds of image variations such as pose may play a more important role than others in achieving image-invariant face recognition. For example, multiple pose encoding has greater power of generalisation to image variation due to illumination, whereas multiple-illumination encoding creates more limited generalisation (Liu et al., 2009). Pose training also transfers better to a new expression, whereas expression training has limited transfer to a new pose (Chen & Liu, 2009). The present study shows that coding different expressions of a face is likely to be more effective than coding a single expression when the observer aims to achieve the best performance for transferring the learning experience to a previously unseen expression. Although these systematic manipulations of image variation help to provide specific predictions about the outcome of face training, recent research has shown that studying the effect of exposure to ‘ambient’ variations that represent the full range of natural variability in images in face stimuli can also reveal important insights about face recognition in more naturalistic settings (e.g., Jenkins, White, Van Montfort & Burton, 2011; White et al., 2014).

The experiments demonstrate that between-expression transfer can be improved after repeated exposures to several different expressions. It should be noted, however, that exposure to a far greater level of image variation is likely to be required to achieve expression-invariant recognition. The clearest effect in all three experiments was the impairment of recognition when studied faces showed a new expression. This was always the case regardless of whether three or one expression was trained.

Greater transfer can be expected from a well-learned single image or a set of images with variations. Past research has shown that repeated exposures to a single image can transfer to new poses or illuminations (e.g., Moses, Edelman & Ullman, 1996; Jiang, Blanz & Tolle, 2007; Longmore et al., 2008, 2014; Roark, O’Toole & Abdi, 2006). The present study shows that repeated exposures to a variety of expressions can transfer more effectively to a new expression than the same level of exposures to a single expression. However, due to the greater amount of information in the multiple-expression training condition, more processing time is likely to be required in our experiments. Because the same amount of time was provided whether the participant had to learn three or one expression, each image in the three-expression condition was likely to be learned less well than the single-expression conditions. The cost of this was reflected in the face-name matching performance of Experiments 2 and 3, where participants made more errors when they had to pair a name with three expressions of a face instead of one. The cost was also reflected in the results of identical expression test condition (Experiment 3), where recognition in the single-expression condition was nearly significantly better than the multiple-expression condition. However, the benefit of studying the expression variation is more evident than the small cost when a studied face was tested with a new expression.

In this study, we have focused on the issue of between-expression transfer without studying whether transfer between some expressions is easier than others. Hence we cannot tell, for example, whether transfer is easier between an emotional and a neutral expression or between two emotional expressions. Transfer between an emotional and a neutral expression has been investigated in a number of studies (e.g., D’Argembeau, van der Linden, Comblain & Etienne, 2003; Baudouin, Gilibert, Sansone & Tiberghien, 2000; Savaskan et al., 2007), but so far few have examined transfer between emotional expressions. Results in our Experiment 3 appear to show that transfer from an emotional face to another emotional one may be easier than from a neutral face to an emotional one. However, it is not possible to draw this conclusion from our testing conditions because the transfer effect required exposure to several expressions, and we did not have a condition in which a neutral expression is also included in the training set. Another unexamined question is whether the power of between-category transfer may be uneven across different categories of expression. Several studies have shown that relative to some other emotional faces, a happy face tends to generalise better to a neutral face (D’Argembeau et al., 2003; Baudouin et al., 2000; Savaskan et al., 2007). However, few studies have tested generalisation from one emotional expression to another emotional one. For example, can an expression of disgust generalise better to an angry expression relative to other expressions? Questions like this will await future research.

Finally, given the important role of non-rigid motion in face recognition (see a review by O’Toole, Roark & Abdi, 2002), comparing effects of static and moving faces should further advance understanding of the learning process toward expression-invariant face recognition. For example, is learning three expressions in video clips more effective than learning the three expressions in static images? Is non-rigid motion of facial expressions more resilient to pose variation (Watson, Johnston, Hill & Troje, 2005)? These remain some of the important outstanding issues. We hope our present findings help future investigations by delineating both the limits and minimal benefits of exposures to a small amount of expression variation under specific static and frontal pose conditions.
